# Semen dysbiosis—just a male problem?

**DOI:** 10.3389/fcimb.2022.815786

**Published:** 2022-09-13

**Authors:** Emilia Morawiec, Michał Czerwiński, Anna Bednarska- Czerwińska, Andrzej Wiczkowski

**Affiliations:** ^1^ Department of Microbiology, Faculty of Medicine, University of Technology in Katowice, Katowice, Poland; ^2^ Gyncentrum Sp. z o.o. Laboratory of Molecular Biology and Virology, Katowice, Poland; ^3^ Department of Histology, Cytophysiology and Embryology, Faculty of Medicine, University of Technology in Katowice, Katowice, Poland; ^4^ American Medical Clinic, Katowice, Poland; ^5^ Faculty of Medicine, University of Technology in Katowice, Katowice, Poland

**Keywords:** semen, microbiome, dysbiosis, infertility, sequencing

## Abstract

Seminal microflora is crucial to male fertility. Dysbiosis—disturbance of quantitative ratios of individual bacteria or appearance of pathogenic species—rarely results in symptomatic disease. Inflammation results in decreased sperm production, lower motility, or morphological changes and, in the long term, can cause ejaculatory duct obstruction, leading to infertility. Moreover, it may cause infection of the partner’s female genital tract. Dysbiosis in both partners results in fertility problems, disorders in embryo implantation, or miscarriages. In addition, chronic inflammation of the male genitourinary system may accelerate the appearance of antisperm antibodies. A comprehensive examination of seminal microflora can clarify the causes of infertility or prevent pathological conditions that affect seminal parameters. Seminal microflora as a direct impact on fertility problems as well as a decrease in the effectiveness of assisted reproduction methods, insemination, or *in vitro* procedures.

## Introduction

Semen is usually examined bacteriologically only when genitourinary infection is suspected and the results of standard physical and biochemical examination indicate it (Dohle et al., 2005; Salonia et al., 2021). Initially, it was believed that the urine and semen of a healthy man were free of bacteria or that bacteria are present only occasionally (Tomaiuolo et al., 2020). Bacteriological examination of the semen was reduced to the classical cultivation of microorganisms in aerobic conditions and, rarely, in anaerobic conditions. Semen, which is a mixture of sperms and accessory gland secretions, contains various nutrients and is therefore an ideal environment for the growth of bacteria (Tomaiuolo et al., 2020). One of the main obstacles to characterizing seminal microbiome was a requirement of specific cultivation conditions and lack of effective cultivation methods.

Molecular diagnostics allowed the detection of numerous bacteria and viruses in the urine and semen of healthy men. In particular, recent advances in next-generation sequencing, which do not require classical culture, have made it possible to characterize the microbiome, including non-culturable species, in various areas of the human body, such as sperm microbiome (Hou et al., 2013; Zhang et al., 2015; Lundy et al., 2021). There are currently three main metagenomic sequencing techniques in use in microbial research: 16SrRNA sequencing, whole-genome sequencing, and metagenomic (shotgun) sequencing. 16SrRNA sequencing is a molecular tool aimed at analyzing the highly conservative sequence of ribosomal 16S and is used to identify different species of bacteria. Modifications of these methods are used to study the genomes of viruses, bacteria, eukaryotes, and metabolic profiles (Hou et al., 2013; Zhang et al., 2015; Koedooder et al., 2019).

The use of sequencing methods in semen analysis revealed that human semen is not sterile and contains specific flora whose functions have not yet been fully understood (Tomaiuolo et al., 2020). In addition, the origin of these microorganisms is unclear. Comparison of bacterial communities detected by 16SrRNA sequencing in semen, urine, and rectal swabs of healthy men showed that only 2.3% of the identified taxa were common to these three environments and 10% to semen and urine (Lundy et al., 2021). However, sperm microbiome is widely believed to originate from infections arising from the urinary tract, blood, and partly from the intestines and vagina. This is indicated by a smaller variation in the microflora composition in men before and after intercourse (Mändar et al., 2015; Restrepo Arenas et al., 2021). Considerable interindividual variability also exists depending on the environment, hygiene, and age (Ma and Li, 2019). A pyrosequencing study by Hou et al. identified 21 genera of bacteria: *Ralstonia*, *Corynebacterium*, *Lactobacillus*, *Streptococcus*, *Staphylococcus*, *Prevotella*, *Finegoldia*, *Anaerococcus*, *Peptoniphilus*, *Incertae Sedis XI*, *Veillonella*, *Pelomonas*, *Porphyromonas*, *Uma*, *Acopidoblas*, *Acopidoblasov*, *Aerococcus*, *Gemella*, *Granulicatella*, *Clostridiales*, and *Cloacibacterium* (Hou et al., 2013). Many of them are anaerobic bacteria and are also found in the vagina. Hou et al. also observed that the species composition of bacterial communities in semen samples varies significantly, suggesting the existence of unique, personalized communities.

## Microbiota profile and fertility

Altmäe et al. demonstrated evidence of the implications of seminal microbiota composition on male reproductive health, couple health, and even offspring health due to the transmission of microorganisms to partners and offspring (Altmäe et al., 2019). Seminal microbiota testing should include the following:

- Unique seminal microbiota—it is necessary to study its composition and function to understand its role in health and disease;
- Changes in the composition of seminal microflora, which is associated with various disorders such as infertility, poor sperm quality, or genital tract inflammation;
- Potential impact of seminal microbiota on partner and offspring health and the course of pregnancy; and
- Considering the microbiota of the partner’s genital tract as well as the couple’s sexual activity.
Research on seminal microbiota is still in its infancy and requires well-designed cohort studies (Altmäe et al., 2019).

According to the World Health Organization (WHO) data, infertility affects approximately 15% of couples trying to have children. In 20%–50% of all cases, decreased male partner fertility is responsible for difficulties in procreation (Kumar and Singh, 2015; Katz et al., 2017; Thoma et al., 2021). Male genitourinary tract infection is believed to be one of the most serious causes of male infertility (Hou et al., 2013; Tomaiuolo et al., 2020).

**Figure 1:**
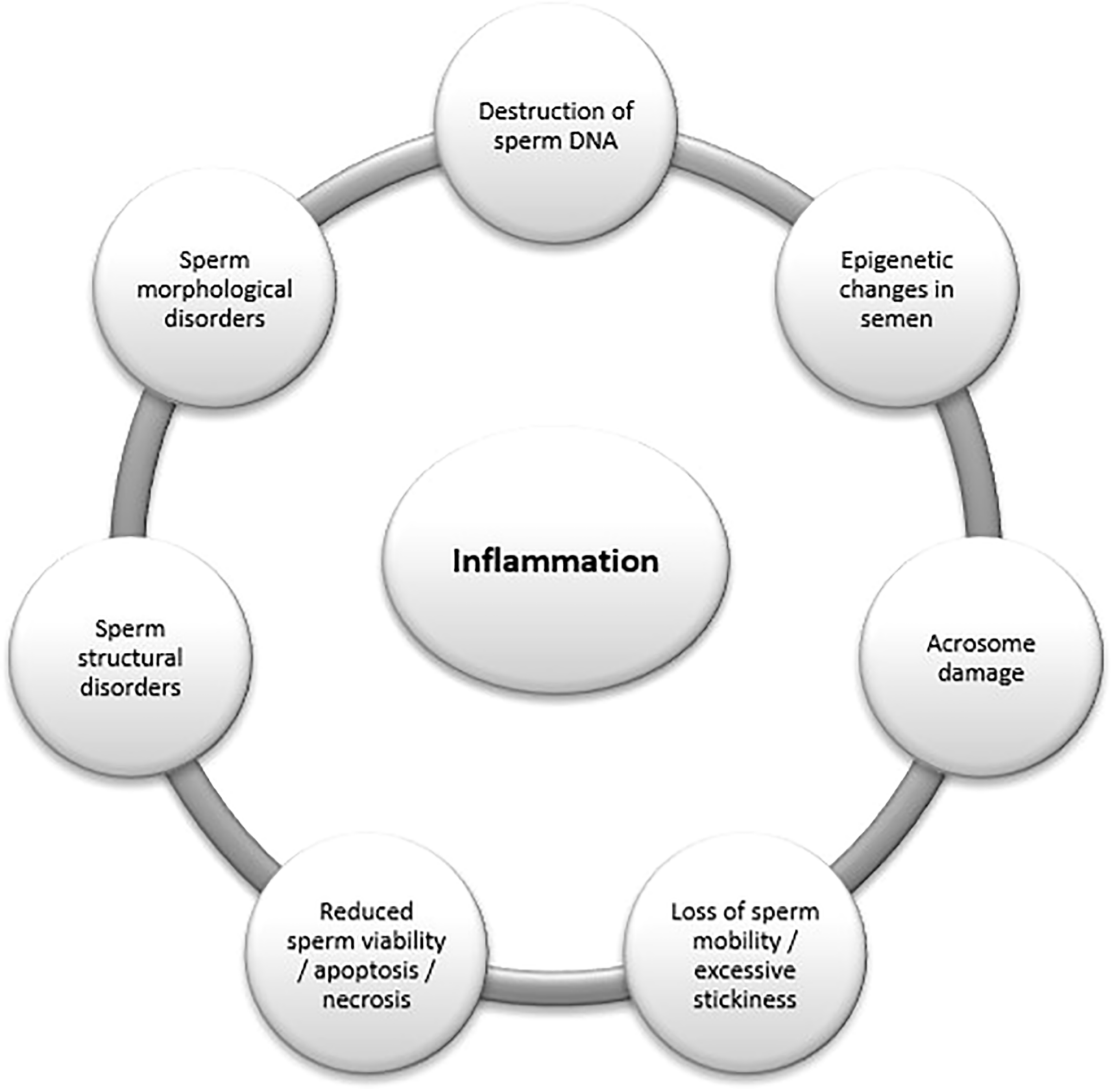
Influence of dysbiosis on semen parameters.

Clinical observation shows that local inflammation in men is usually asymptomatic, and its effects only become apparent after a long time (Dohle et al., 2005; Zhang et al., 2015). Infection of any of the various male reproductive system organs causes local inflammatory processes, which can cause anatomical changes in the genital tract and affect the structure and function of semen spermatozoa (Koedooder et al., 2019) (**Table 1**) (**Figure 1**). The clinical significance of bacteriospermia and its effect on the reduction of fertility are well known. Pathological changes in the semen may result from direct or indirect damage to sperms and the process of spermatogenesis through cytokines and free radicals formed during inflammatory reactions, as well as toxins and bacterial enzymes (Tomaiuolo et al., 2020; Lundy et al., 2021). These changes include dysfunction of the accessory glands; inflammation-induced obstruction of semen transport; disorders of spermatogenesis by direct action of pathogens or their components and/or induction of cellular and humoral immune responses with disruption of specific local immune regulation in testes; induction of a humoral response directed against sperm; impaired sperm motility; disturbance of acrosomal response and normal sperm morphology; pathogen-induced epigenetic changes; and sperm DNA fragmentation (Hou et al., 2013; Mändar et al., 2015; Koedooder et al., 2019; Lundy et al., 2021; Restrepo Arenas et al., 2021). These negative changes are usually associated with infection with bacteria such as *Escherichia coli*, *Staphylococcus aureus*, *Enterococcus faecalis*, *Klebsiella* sp., *Streptococcus* sp., *Chlamydia trachomatis*, *Ureaplasma urealyticum*, and *Mycoplasma hominis* (Hou et al., 2013; Mändar et al., 2015; Lundy et al., 2021; Restrepo Arenas et al., 2021). However, there are doubts about the clinical role of opportunistic flora detected on the basis of sequential analyzes in ejaculates, which colonize and contaminate the male genitourinary system.

**Table 1:** Observations related to the condition and quality of semen caused by the presence of specific bacterial species in healthy patients and in those with pathologies.

Observation	Flora	Reference
**Physiology**		
Study of the control group	*Pelomonas, Propionibacterium, Bosea, Afipia, Sphingomonas, Vogasella, Brevibacillus, Xylanimicrobium, Flexispira, Pedomicrobium, Phyllobacterium, Aquimonas, Dietzia, Sediminibacterium, Mycobacterium, Eikenella, Brevibacterium, Corynebacterium, Eubacterium*, and *Bacillus* sp.	(Yang et al., 2020)
Good-quality semen	*Lactobacillus*	(Tomaiuolo et al., 2020)
Normospermia	*Lactobacillus* and *Gardnerella domination*	(Okwelogu et al., 2020)
Maintaining good semen quality, protection against the negative impact of Gram-negative bacteria.	Higher ratio of *Propionibacterium* spp. and *Atopobium* spp.	(Weng et al., 2014)
**Pathology**		
Low sperm quality/ Flora of men with infertility	Prevotella/ *Lactobacillus*, *Pseudomonas*, *Prevotella*, *Gardnerella*, *Rhodanobacter*, *Streptococcus*, *Finegoldia*, and *Haemophilus*	(Weng et al., 2014)
Flora of men with impaired fertility (asthenozoospermia and oligoasthenozoospermia), decreased sperm motility	*Lactobacillus*, *Bacteroides*, *Delftia*, *Sneathia*, *Enhydrobacter*, *Anaerococcus*, *Mycoplana*, *Finegoldia*, *Stenotrophomonas*, *Methylobacterium*, *Coprobacillus*, *Aerococcus*, *Atopobium*, *Chryseobacterium*, *Kocuria*, *Megasphaera*, *Ralstonia*, *Achromobacter*, *Ervinia*, *Ureaplasma*, *Filifactor*, *Prevotella*, *Saccharopolyspora*, and *Porphyromonas*	(Yang et al., 2020)
Low-quality semen, decreased sperm motility, abnormal sperm morphology	*Anaerococcus*, *Bacteroides ureolyticus*, *Proteobacteria*, and *Prevotella*	(Tomaiuolo et al., 2020)
Azoospermia	*Bacteroides* and *Firmicutes*	(Tomaiuolo et al., 2020)
Pathological changes in semen.	*Haemophilus haemolyticus*, *Haemophilus parainfluenzae*, *Enterococcus faecalis*, *Gardnerella vaginalis*, *Escherichia coli*, *Streptococcus anginous*, and *Streptococcus agalactiae*	(Pagliuca et al., 2021)
Abnormal semen parameters	*Prevotella* domination	(Baud et al., 2019)
Azoospermia	Predominance of *Bacteroides* and *Firmicutes*, less flora diversity	(Chen et al., 2018)
Inflammation	*Gardnerella*	(Okwelogu et al., 2020)
Low sperm motility	*Corynebacterium* domination	(Farahani et al., 2021)
Sperm necrosis and oligozoospermia	*Streptococcus anginosus*	(Lundy et al., 2020)
Indicator of poor sperm quality, higher ratio is associated with infertility, reduced sperm motility and their abnormal morphology	*Anaerococcus hydrogenalis*	(Koedooder et al., 2019)
Negatively affects sperm number, sperm motility and affects their morphology	*Mycoplasma hominis*	(Koedooder et al., 2019; Farahani et al., 2021)
Excessive semen viscosity and OAT	Increase in *Neisseria or Klebsiella pneumoniae*, reduction of *Lactobacillus*	(Monteiro et al., 2018; Koedooder et al., 2019)
Low sperm motility, acrosome damage, sperm DNA fragmentation, sperm death	*Escherichia coli*, *Proteus mirabilis*, and *Proteus vulgaris*	(Bhatt et al., 2015)
Negative effect on semen quality, reduction of sperm motility and quantity with abnormal morphology	*Pseudomonas aeruginosa* and *Pseudomonas putida*	(Fraczek and Kurpisz, 2015)
Increased apoptosis and necrosis of sperm in semen	*Morganella morganii*	(Moretti et al., 2009)

^*^OAT Excessive sperm viscosity and oligoasthenoteratozoospermia.

Hou et al. (2013) found no significant differences in the composition of the control group’s flora compared with men with infertility. Only a correlation between reduced semen quality and the presence of *Anaerococcus* in men with infertility was demonstrated (Hou et al., 2013). Weng et al. analyzed samples of 96 men with infertility and found the presence of *Lactobacillus*, *Pseudomonas*, *Prevotella*, *Gardnerella*, *Rhodanobacter*, *Streptococcus*, *Finegoldia*, and *Haemophilus*, with *Prevotella* occurring more frequently in people with low sperm quality (Weng et al., 2014). Yang et al. included 101 men with dysspermia and 58 healthy men and showed a significant difference between the composition of seminal microbiota of healthy men and microbiota of people with asthenozoospermia and oligoasthenozoospermia (Yang et al., 2020). The following bacteria have been found in patients with dysspermatism: *Lactobacillus*, *Bacteroides*, *Delftia*, *Sneathia*, *Enhydrobacter*, *Anaerococcus*, *Mycoplana*, *Finegoldia*, *Stenotrophomonas*, *Methylobacterium*, *Coprobacillus*, *Aerococcus*, *Atopobium, Chryseobacterium*, *Kocuria*, *Megasphaera*, *Ralstonia*, *Achromobacter*, *Ervinia*, *Ureaplasma*, *Filifactor*, *Prevotella*, *Saccharopolyspora*, and *Porphyromonas.* However, in the healthy control group, the dominant types were *Pelomonas*, *Propionibacterium*, *Bosea*, *Afipia*, *Sphingomonas*, *Vogasella*, *Brevibacillus*, *Xylanimicrobium*, *Flexispira*, *Pedomicrobium*, *Phyllobacterium*, *Aquimonas*, *Dietzia*, *Sediminibacterium*, *Mycobacterium*, *Eikenella*, *Brevibacterium*, *Corynebacterium*, *Eubacterium*, and *Bacillus* sp. In men with asthenozoospermia and oligozoospermia, gram-negative species prevailed, providing lipopolysaccharides and stimulating inflammatory reactions, which may cause sperm DNA destruction (Yang et al., 2020). For example, *Escherichia coli* immobilizes sperm by direct contact and destroys their morphology. In this process, pili, type-1 fimbriae, P fimbriae, and mannose receptor-dependent interactions may be involved. The pathogenic effect of microorganisms on sperm results from not only the close contact of cells with bacteria but also the influence of virulent factors, including lipopolysaccharides, cytotoxic necrotizing factor, hemolysins, and sperm immobilization factor. Hemolysins may disrupt the integrity of the cell membrane, and sperm immobilization factor reduces the activity of mitochondrial ATPase, thus reducing sperm mobility and viability (**Figure 1**) (Fraczek and Kurpisz, 2015; Oghbaei et al., 2020).

In men with genitourinary infections, long-term antibiotic therapy led to an improvement in semen parameters, including sperm morphological abnormalities (Menkveld, 2004; Pasquale et al., 2021). Antibiotic therapy increases sperm concentration and significantly reduces sperm DNA fragmentation in men with urogenital infection and statistically (**Figure 1**) (Bezold et al., 2007; Ahmadi et al., 2018; Eini et al., 2021). Tomaiuolo et al. concluded that the presence of *Lactobacillus* accompanies good-quality semen. On the other hand, *Anaerococcus*, *Bacteroides ureolyticus*, *Proteobacteria*, and *Prevotella* were more commonly detected in low-quality semen samples, and *Bacteroides* and *Firmicutes* in azoospermic semen (Tomaiuolo et al., 2020). Pagliuca et al. divided 53 men into two groups depending on the presence or absence of changes in their semen. In the group with changes, 70.2% showed microorganism growth, and bacteria belonged to 18 species, with a clear predominance of coagulase-negative staphylococci. *Haemophilus haemolyticus*, *Haemophilus parainfluenzae*, *Enterococcus faecalis*, *Gardnerella vaginalis*, *Escherichia coli*, *Streptococcus sangineous*, and *Streptococcus agalactiae* were observed. Presence of bacteria in the semen was also significantly negatively correlated with semen volume, sperm count, and sperm motility (Pagliuca et al., 2021).

Similarly, Baud et al. examined the semen of 94 men using 16SrRNA sequencing, including 26 with normal spermiogram and 68 with abnormal parameters such as volume, number, motility, and morphology (Baud et al., 2019). They found three groups based on bacterial predominance: *Prevotella* predominance, *Lactobacillus* predominance, and a mixed group with no significant differentiation, with numerous representations of *Staphylococcus* and *Corynebacterium*. *Prevotella* was more often detected in the group with impaired sperm motility and *Lactobacillus* in samples with normal morphology; *Staphylococcus* was present more frequently in the control group. Baud et al. stated that the total number of bacteria may not play a major role in male infertility. They also did not demonstrate a significant effect of microbial groups on semen quality but suggested that some types of bacteria may affect sperm motility and morphology (Baud et al., 2019). The presence of *Lactobacillus* in semen of healthy men was also demonstrated by Chen et al. (2018). They studied three groups of men: men with normal semen, those with obstructive azoospermia, and those with unobstructive azoospermia. Compared with the normal semen group, men with azoospermia showed less diversity of flora but had a predominance of *Bacteroides* and *Firmicutes*. *Proteobacteria* and *Actinobacteria* were more common in the normal semen group. Some *Firmicutes* bacteria produce endospores, making them resistant to drying out and able to survive in extreme environmental conditions. The *Bacteroides* cluster contains three large classes of gram-negative, aerobic, and anaerobic bacteria, several of which are opportunistic. An increase in the number of species of these two clusters in patients with azoospermia may also pose a risk of gynecological inflammation in their partners (Chen et al., 2018).

Farahani et al. observed that a marked increase in the number of *Corynebacterium*, which is part of the natural microbiota, reduces sperm motility (Farahani et al., 2021). Weng et al. noted a higher ratio of *Propionibacterium* spp. and *Atopobium* spp. in normal semen compared with abnormal samples and concluded that these bacteria are involved in maintaining good semen quality and can protect against the negative effects of gram-negative bacteria (Weng et al., 2014). Lundy et al. demonstrated the presence of *Streptococcus anginosus* in semen of fertile and men with infertility. In the case of men with infertility, increased sperm necrosis and a decrease in their number were observed (Lundy et al., 2020). Excessive sperm viscosity and oligoasthenoteratozoospermia (OAT) have been associated with an increase in *Neisseria* or *Klebsiella pneumoniae* and a decrease in *Lactobacillus* in semen (Monteiro et al., 2018; Koedooder et al., 2019). The presence of *Escherichia coli*, *Proteus mirabilis*, or *Proteus vulgaris* has been associated with reduced sperm motility, acrosome damage, DNA fragmentation, or death (Bhatt et al., 2015). Other pathogenic species found in semen of men with infertility were *Pseudomonas aeruginosa* or *Pseudomonas putida*, whose presence was associated with a reduction in the amount and occurrence of sperm with abnormal morphology. Moreover, the imbalance of *Pseudomonas* to the detriment of *Lactobacillus* has been associated with excessive semen viscosity and OAT (Fraczek and Kurpisz, 2015). Increased apoptosis and necrosis of sperm were reported in semen in which *Morganella morganii* was also present (**Figure 1**) (Moretti et al., 2009).

## Microflora of partners

A close relationship exists between the microbiota of sexual partners, and intercourse leads to the exchange of microorganisms (Tomaiuolo et al., 2020). Swanson et al. claim that 85% of species of semen bacteria also present as vaginal microbiota (Swanson et al., 2020). Mändar et al. and Okwelogu et al. on couples applying for *in vitro* fertilization showed that despite the smaller number of sperm microbiota, there is a great similarity with the vaginal microbiota of sexual partners. At the same time, the studied semen had high species diversity. *Lactobacillus* and *Gardnerella* were dominant in semen of men with normospermia. In women, in addition to *Lactobacillus*, *Prevotella* and *Gardnerella* were shown to be present. The authors suggest an unfavorable effect of *Gardnerella* in men with fertilization problems (Mändar et al., 2015; Mändar et al., 2018; Okwelogu et al., 2020). These studies support the hypothesis that female genital microbiota may affect male microbiota. In a study of 53 couples with idiopathic infertility, Amato et al. found a very diverse flora in male semen, including species also found in their partners’ vaginas, but did not observe a statistical relationship with semen parameters that were within the normal range. In women, a diverse composition of species of the genus *Lactobacillus* was demonstrated, which may be important for the success of intrauterine insemination (Amato et al., 2020; García-Velasco et al., 2020). The presence of *Lactobacillus crispatus* was an indicator of a healthy vaginal ecosystem, whereas *Lactobacillus iners* and *Lactobacillus gasseri* indicated a dysbiotic environment (Amato et al., 2020; Okwelogu et al., 2020). The microbiota continuum in the female genital tract was demonstrated by Chen et al. (2017). Female microbiota creates a non-sterile environment that varies from vagina to ovaries, depending on menstrual cycle, age, and health. Both the upper and lower genital tracts are (unlike the vagina) periodic places of colonization by anaerobic and aerobic bacteria. *Lactobacillus* predominates in the vagina and cervix, and mainly *Pseudomonas*, *Vagococcus*, *Acinetobacter*, *Sphingobium*, *Comomadaceae*, and nine other groups of bacteria have been detected in rectouterine pouches. Similarly, in the uterus, in addition to *Lactobacillus*, *Pseudomonas*, *Vagococcus*, *Sphingobium*, *Comomada*, and companion bacteria, occurring in small amounts, have also been found. *Acinetobacter* and *Vagococcus* are dominant in the fallopian tubes, and 20 other types of microorganisms have been found (Chen et al., 2017). Jankowska et al. and Carda-Diéguez et al. also indicate the relationship between the semen flora and genital tract of female partners, emphasizing that exchange of flora may lead to dysbiosis or inflammation. Treatment with antibiotics cannot always restore homeostasis and may affect the physiology of gametes or embryos (Carda-Diéguez et al., 2019; Jankowska et al., 2020; Pagliuca et al., 2021). These observations support the hypothesis about the influence of paternal microbiota on epigenetic changes in fetuses and the course of pregnancy (Altmäe et al., 2019). For example, Wittermer et al. analyzed 951 couples undergoing *in vitro* fertilization and demonstrated dysbiotic cultures of semen, vaginal, and cervical swabs in 77 pairs. The percentage of clinical pregnancies in this group was 19.5% compared with 36.2% in cases of vaginal infection only (Wittemer et al., 2004). The role of epigenetic mechanisms related to the influence of sperm microflora is indicated by Rando et al. in their work on the impact of parents’ diet on the metabolism of offspring (Amato et al., 2020). On the other hand, Pan et al. suggest an influence of sperm microbiome on the transcriptome and DNA methylation signature. They observed that the influence of bacteria on transcriptome increased over time, but they detected the most microbiota-dependent differences immediately after birth, which indicates an earlier influence of both maternal and paternal bacteria (Pan et al., 2018).

## Concluding remarks

The present review of recent studies on the seminal microbiome by using sequence analysis revealed that there is a great diversity of microorganisms in the male genital tract. However, there is no clear definition of the importance of individual groups and the proportion of bacteria in infertility. Those who conduct research on seminal microbiota must define which of these microorganisms are tourists, residents, and invaders (Altmäe et al., 2019). Previous culture-based studies have shown a significant association of bacteriospermia with leukospermia and pathological results of semen studies, as indicated by macroscopic, microscopic, and physicochemical changes. The inflammatory response to infection was illustrated by leukospermia, free radical activity, and DNA defragmentation (La Vignera et al., 2014; Lundy et al., 2021; Salonia et al., 2021). Research has revealed sets of different bacterial species in various parts of the genital tract, which may be associated with different disease states. Their origin is diverse; some of them come from the urinary tract, some from the gastrointestinal tract, and others change depending on age, sexual activity, sanitary habits, and characteristics of the microorganisms themselves, such as the ability to form biofilm (Mändar et al., 2015; Altmäe et al., 2019; Lundy et al., 2021). Much research attention is focused on identifying bacteria with undetermined influence on semen quality, i.e., *Actinobaculum* (Actinotignum) *urinale* (Fendukly and Osterman, 2005; Lotte et al., 2016), *Actinomyces radingae* (Clarridge and Zhang, 2002; Lotte et al., 2016), *Mobiluncus* spp. (Lotte et al., 2016), *Acidovorax* spp. (Hou et al., 2013), *Aeromonas* spp. (Gonçalves Pessoa et al., 2019), *Aggregatibacter* spp. (Monteiro et al., 2018), or *Acinetobacter johnsonii* (Koedooder et al., 2019). These results indicate that colonization applies to all spaces that can be inhabited and that some of these microorganisms are involved in the induction of inflammation. However, diverse flora may be necessary for properly functioning semen and protecting against pathogenic bacteria, as in the case of vaginal microflora (Farahani et al., 2021). Numerous studies have shown interactions between female and male microbiota; therefore, the diagnosis of dysbiosis as the cause of infertility should always include both sexual partners.

## Author contributions

The manuscript was equally drafted, revised, and approved by AW, EM, MC, and AB-C. All authors contributed to the article and approved the submitted version.

## Conflict of interest

EM was employed by the company Gyncentrum.

The remaining authors declare that the research was conducted in the absence of any commercial or financial relationships that could be construed as a potential conflict of interest.

## Publisher’s note

All claims expressed in this article are solely those of the authors and do not necessarily represent those of their affiliated organizations, or those of the publisher, the editors and the reviewers. Any product that may be evaluated in this article, or claim that may be made by its manufacturer, is not guaranteed or endorsed by the publisher.
